# Recent Advances in Glycyrrhetinic Acid-Functionalized Biomaterials for Liver Cancer-Targeting Therapy

**DOI:** 10.3390/molecules27061775

**Published:** 2022-03-08

**Authors:** Antonio Speciale, Claudia Muscarà, Maria Sofia Molonia, Mariateresa Cristani, Francesco Cimino, Antonella Saija

**Affiliations:** Department of Chemical, Biological, Pharmaceutical and Environmental Sciences, University of Messina, Viale Ferdinando Stagno D’Alcontres 31, 98166 Messina, Italy; specialea@unime.it (A.S.); cmuscara@unime.it (C.M.); mmolonia@unime.it (M.S.M.); mcristani@unime.it (M.C.); asaija@unime.it (A.S.)

**Keywords:** glycyrrhetinic acid, liver cancer, liver targeting, drug delivery systems

## Abstract

Liver cancer is one of the most common causes of cancer mortality worldwide. Chemotherapy and radiotherapy are the conventional therapies generally employed in patients with liver tumors. The major issue associated with the administration of chemotherapeutics is their high toxicity and lack of selectivity, leading to systemic toxicity that can be detrimental to the patient’s quality of life. An important approach to the development of original liver-targeted therapeutic products takes advantage of the employment of biologically active ligands able to bind specific receptors on the cytoplasmatic membranes of liver cells. In this perspective, glycyrrhetinic acid (GA), a pentacyclic triterpenoid present in roots and rhizomes of licorice, has been used as a ligand for targeting the liver due to the expression of GA receptors on the sinusoidal surface of mammalian hepatocytes, so it may be employed to modify drug delivery systems (DDSs) and obtain better liver or hepatocyte drug uptake and efficacy. In the current review, we focus on the most recent and interesting research advances in the development of GA-based hybrid compounds and DDSs developed for potential employment as efficacious therapeutic options for the treatment of hepatic cancer.

## 1. Introduction

Liver cancer is one of the most common cancers and among the most common causes of cancer mortality worldwide [1]. The highest percentage of primary liver cancers is represented by hepatocellular carcinoma (HCC) [2]. Since surgical resection is feasible in only a few patients, chemotherapy and radiotherapy are the conventional therapies generally employed in patients with liver tumors. Various chemotherapeutic agents, including doxorubicin (DOX), mitoxantrone, gemcitabine, irinotecan, sorafenib, etc., used both as single or as combinational agents, are available for the treatment of HCC [3,4]. Furthermore, a number of novel therapeutic strategies, such as treatments targeting cancer stem cells, molecular targeted therapy, and immunotherapy, are going to be developed [5,6]. However, poor prognosis is found in the majority of patients with HCC due to characteristics intrinsic to the tumor (in particular drug resistance) and mainly due to poor drug bioavailability, non-selective biodistribution, low specificity, and systemic toxicity.

An enormously important approach to the development of original therapeutic products actively targeting liver cancer takes advantage of the employment of biologically active ligands able to bind specific receptors on cytoplasmatic membranes of liver cells. In fact, these ligands may be used to synthesize hybrid compounds based on traditional chemotherapy drugs or to functionalize the surface of micro- and nano-drug delivery systems (DDSs) since they can allow a specific and efficient drug internalization into tumoral cells [7].

In particular, regarding DDSs, more extensive angiogenesis is typical of tumors since more oxygen and nutrients are needed for their rapid growth [8]. However, tumor vasculature is dramatically different from that in normal tissue, with the presence of discontinuous endothelial lining and fenestrations and a lack of smooth muscle cells and pericytes [9]. A much higher DDS accumulation in tumor mass than in normal tissues is an effect known as the enhanced permeability and retention (EPR) effect, which mainly depends on the size of the DDS. On the one hand, the fenestrations in the liver sinusoidal endothelium facilitate substrate transfer into space of Disse between the liver sinusoid and hepatocytes in a normal liver. On the other hand, a longer circulation time of the nanosystems, achieved by opportune surface functionalization strategies, is also extremely important for their accumulation into tumor tissues [10,11]. Since DDSs accumulated into tumor interstitial fluid may be specifically internalized into tumor cells due to specific cell surface interactions, receptor-mediated endocytosis is an approach to active drug delivery targeted to liver cancer cells. In fact, some proteins and molecules are overexpressed on the surface of hepatoma cells or tumoral vessels, and their ligands (polysaccharides, vitamins, peptides, aptamers, transferrin, growth factors, etc.) may be utilized to functionalize DDSs to be specifically recognized by the tumor cells. Following cell internalization through receptor-mediated endocytosis, the drugs loaded in targeted DDSs (TDDSs) are released into the cytoplasm.

The *Glycyrrhiza* genus (Fabaceae family), also known as licorice, is extensively spread in the Mediterranean basin of Africa, Europe, and Asia, and *Glycyrrhiza glabra* L. is commercially the most important species belonging to this genus, largely employed as food and for medicinal purposes. Glycyrrhetinic acid (GA), also known as enoxolone, is a triterpenoid derivative of beta-amyrin and is the aglycone derived from intestinal hydrolysis of glycyrrhizin, a pentacyclic triterpenoid present in roots and rhizomes of licorice [12]. GA occurs naturally as 18β-GA, derived from 18β-glycyrrhizin, and may be isomerized into the α-isoform under alkaline conditions [13]. It is reported to have hepatoprotective activity but also demonstrates anticancer ability against HCC by multiple mechanisms, including inhibition of cell proliferation, invasion and metastasis, cell cycle arrest, induction of autophagy and apoptosis, and reduction of immunosuppression [14]. 

GA has been used as a ligand for liver targeting due to the expression of GA receptors on the sinusoidal surface of mammalian hepatocytes, so it may be employed to modify DDSs and obtain better liver or hepatocyte drug uptake and efficacy [15,16]. Previous studies have demonstrated that GA could bind with high affinity to cytomembrane-localized receptors in hepatocytes, and these proteins were named “GA receptors” [15,17]. Recently, Sun and colleagues further confirmed the competitive binding of fluorescein isothiocyanate-GA (FITC-GA) and GA to these receptors in HCC cells [18]. It has been demonstrated that the C11-carbonyl and C3-hydroxyl groups of GA have limited influence on the targeting action of GA to HCC cells and that the β-configuration hydrogen atom at the C18 position of GA contributes the most targeting effect [19]. It is believed that potential GA receptor-mediated hepatic targeting of GA is critical for the anti-HCC effects of GA. GA receptors are predominantly expressed in the liver but not in other organs [15,20], and liver tumor tissue possesses 1.5- to 5-fold more GA receptors than normal tissues. Some other kinds of receptors could be useful to project active hepatic-TDDSs (such as liposomes, micelles, and nanoparticles), including glycyrrhizin receptor (GL-R), asialoglycoprotein receptor (ASGP-R), hyaluronan receptor (HA-R), folate receptor (FA-R), and epidermal growth factor receptor (EGF-R). However, ASGP-R is normally expressed in hepatoma cells and normal hepatocytes, while data on FA-R expression in HCC are controversial. In addition, targeting transferrin receptor, HA-R, and EGF-R gives unexpected immunogenicity to protein ligands [21,22]. Finally, the GA binding sites bind to more than glycyrrhizin [20].

In the current review, we focus on the most recent and interesting research advances in the development of GA-based hybrid compounds and DDSs developed for potential employment as efficacious therapeutic options for the treatment of hepatic cancer. 

## 2. Antitumoral Effects of Glycyrrhetinic Acid

Since in some delivery systems GA is employed both as a ligand to target HCC cells and as a cytotoxic drug, in this paragraph, we have briefly reviewed the molecular mechanisms underlying GA’s anticancer effects. 

GA exerts a remarkable antitumoral effect against various cancers, including lung cancer, pituitary adenoma cells, glioblastoma cells, and prostate cancer, as well as HCC. By analyzing the anticancer activity of a large number of GA derivatives as related to the structural features, Xu et al. [23] have suggested that the A ring skeleton, the C3-hydroxyl group, and the C30-carboxyl group are critical for GA’s antitumor activity, whereas the C11-keto group does not show a correlation with the cytotoxicity. 

At the cellular level, GA acts, through multiple mechanisms, by inhibiting cell proliferation, invasion, and metastasis and inducing autophagy, apoptosis, and cell cycle arrest [14,15,17,24,25,26,27,28]. Unfortunately, GA has poor water solubility and bioavailability; however, many studies have demonstrated that GA delivery through micro- and nanosystems represents an efficient strategy to ameliorate its bioavailability [15]. Furthermore, numerous GA derivatives with more potent cytotoxicity have been explored [24]. In particular, the conjugation of GA with other anticancer molecules may produce a synergic enhancement of their combined cytotoxicity. 

**Figure 1:**
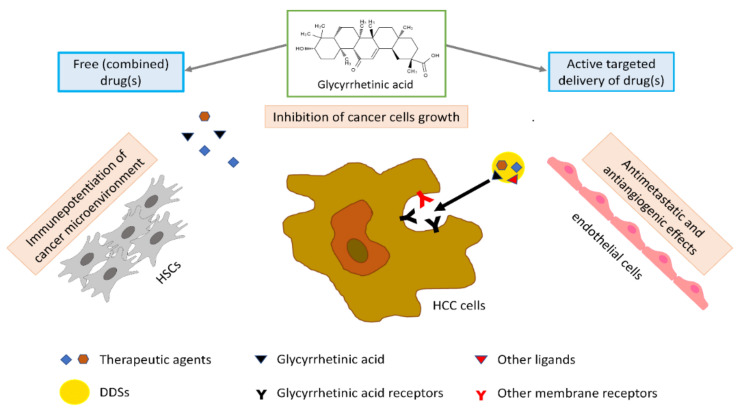
Schematic representation of mechanisms involved in the antitumoral effects of GA used as a free cytotoxic drug or as a ligand to target HCC cells. HCC: hepatocellular carcinoma; HSC: hepatic stellate cell; GA: glycyrrhetinic acid.

More in particular, as to HCC, GA exerts an active anticancer effect by inhibiting cell proliferation, inducing apoptosis, and arresting cell cycle in the G1-phase. The apoptotic effect of GA seems to be related to the activation of caspase-8 and reduction of the antiapoptotic proteins B-cell lymphoma 2 (Bcl-2) and Bcl-xL, with consequent activation of downstream mitochondrial pathways and caspase-3. GA can reverse immunosuppression due to hepatic stellate cells (HSCs) in the tumor microenvironment and abrogate their invasiveness, thus potentiating an immune microenvironment in tumors. The antiangiogenic and antimetastatic effect of GA in HCC could be related to downregulation of the expression of vascular endothelial growth factor (VEGF), lymphatic vessel endothelial hyaluronan receptor 1 (LYVE-1) (a marker for lymphatic metastasis), and matrix metalloproteinase-2 (MMP-2) protein (Figure 1). 

However, there is evidence that the autophagic response induced by GA via ERK (extracellular signal-regulated kinase) activation could weaken its anti-HCC activity. This effect of GA has to be taken into consideration, especially in the case of combinatorial therapy [26,29]. Furthermore, GA was also found to reduce the expression of connexin 32 and actin and inhibit the gap junction [25]; this effect might counteract the cytotoxicity of other antitumoral drugs given in combination and allow cell extravasation and, thus, cancer metastasis [30].

A particularly interesting issue is represented by the research about triterpenoids, as well as other natural compounds, as mitochondria-targeted anticancer drugs. Mitochondria represent the main cellular source of adenosine triphosphate (ATP) produced through oxidative phosphorylation. Under aerobic conditions, to produce ATP, normal cells use the glycolytic pathway to transform glucose into pyruvate in the cytosol; then, pyruvate is converted into acetyl-CoA within the mitochondria. Conversely, under anaerobic conditions, a lower amount of pyruvate is employed to produce ATP, and cytoplasmatic glycolysis is preferred. However, cancer cells show a particular biochemical behavior (“aerobic glycolysis”), since under aerobic conditions, they produce energy mainly through the glycolytic pathway that should be employed to produce the macromolecules needed for biosynthesis; furthermore, in this way, cells produce more lactic acid which has an important role in cancer progression [31]. Besides their role in energy production, mitochondria play a central role in controlling cell apoptosis. Apoptosis is regulated by the extrinsic pathway (also known as the death-receptor pathway) and the intrinsic pathway, or mitochondrial pathway. The latter pathway is activated through changes in mitochondrial outer membrane permeabilization, membrane potential (∆Ψm) collapse, assembly of the permeability pore complex, activation of pro-apoptotic Bcl-2 proteins, release of mitochondrial pro-apoptotic factor cytochrome c (Cyt c) into cytoplasm and activation of caspases (caspase-9, caspase-7, and caspase-3) [32]. As demonstrated by Salvi et al. [33] and Fiore et al. [34], GA induces oxidative stress in liver mitochondria. In fact, GA interaction with the mitochondrial respiratory chain triggers the generation of hydrogen peroxide, which is responsible for the oxidation of critical thiols and pyridine nucleotides, with a consequent opening of the mitochondrial permeability transition pores. However, mitochondria are highly impermeable organelles. Although the outer membrane is non-specifically permeable to all low-molecular-weight solutes, the inner membrane is impermeable, and metabolite exchange across the inner membrane depends on specific transporters. However, under some conditions (in particular stresses), permeability transition pores of the inner membrane open and cause mitochondrial permeability transition (MPT) [35]. Some papers have proven the efficacy of triterpenoids, including GA, when linked to mitochondria-targeted carrier molecules, in particular delocalized lipophilic cations such as triphenylphosphonium derivatives [36,37], since these small molecules easily accumulate into mitochondria because the mitochondrial membrane potential is notably higher than the plasma membrane potential (150–180 mV and 30–60 mV respectively) [38].

## 3. GA-Based Hybrid Molecules

Pharmacophore hybridization is a new strategy for designing and developing new bioactive molecules based on the combination of pharmacophoric moieties of different bioactive compounds by covalent bonds to generate an original hybrid entity with better pharmacokinetic and pharmacodynamic properties in comparison with the parent drugs. Furthermore, this approach can result in compounds exhibiting modified selectivity profiles, different and/or dual modes of action, and reduced side effects [39]. 

**Figure 2:**
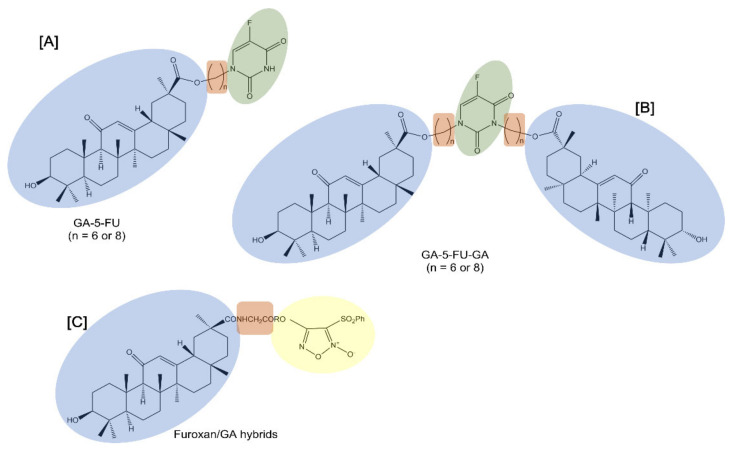
Examples of GA-based hybrid molecules. (**A**) Mono- or (**B**) di-conjugates of GA (in blue) with 5-FU (in green) (GA-5-FU and GA-5-FU-GA, respectively) through alkyl chains as a linker (in the orange box) where n is the number of methylene groups [40]. (**C**) Conjugates of GA (in blue) with furoxan (in yellow) bearing a glycine residue (in the orange box) as a linker [41]. R = (CH_2_)_3_O; (CH_2_)_4_O; CH_2_CH=CHCH_2_O; CH_2_C≡CCH_2_O. GA, glycyrrhetinic acid; 5-FU: 5-fluorouracil.

Notably, 5-fluorouracil (5-FU) is a cell cycle-specific anticancer drug that interferes with DNA synthesis and inhibits RNA formation. Despite its useful activity, 5-FU exhibits evident adverse effects which limit its clinical usefulness. For this reason, structural modification of 5-FU was performed to improve its selectivity and reduce the toxic side effects. Alkyl chains are usually used as the linkers of two pharmacophores in order to change the physical and chemical properties and increase liposolubility [40] (Table 1). In this regard, 5-FU was attached to GA to provide mono- or di-conjugates of the pentacyclic triterpene with 5-FU (GA-5-FU and GA-5-FU-GA respectively) through alkyl chains (6 or 8 methylene groups) (Figure 2). Data showed that the double substitution targeted hybrids did not have antitumor activity, while the single substituted hybrids improved antitumor activity against tested cancer cell lines, such as human HCC Bel-7402 cells. As studied in MDR A549 cells, the GA-5-FU monomer induced changes in intracellular calcium influx and the generation of ROS as a result of significant apoptosis induction.

Similarly, furoxans, an important class of nitric oxide (NO) donors, were derivatized with GA to produce novel furoxan/GA hybrids to selectively target the liver by producing high concentrations of NO in HCC cells and to overcome furoxan’s severe adverse effects [41] (Figure 2). NO, a pleiotropic regulator critical for numerous biological processes, is known to kill a range of tumor cells of different origins and grades through direct and indirect mechanisms [42]. Furoxans/GA hybrids produced high concentrations of NO and exhibited potent and selective cytotoxicity against HCC cells while demonstrating few side effects on healthy hepatocytes. In addition, since coupling with an amino acid can help in delivering the compounds to tumor cells, the introduction of a glycine residue as a linker into compounds significantly enhanced their potent cytotoxicity selectively against HCC cells. In fact, some of these furoxan/GA hybrids with a glycine residue as a linker showed a selective cytotoxic effect that was notably stronger against HCC BEL-7402 and HepG2 cells than against non-tumor human liver LO2 cells.

In order to improve GA solubility and bioavailability, a novel conjugate combining GA, tetramethylpyrazine, and a small-molecule amino acid (TOGA) was studied [43]. TOGA reduced in vitro HepG2 cell migration and invasion accelerated by co-cultured tumor-associated macrophages and mitigated IL-1β-induced HepG2 NF-κB activity, a key pathway associated with tumor progression. In vivo data confirmed TOGA’s antitumor activity since it inhibited tumor volume and weight in HepG2 xenograft nude mice and H22 orthotopic mice models. In addition, TOGA treatment strongly downregulated the expression of IL-1R1, known for its inflammatory responses and immunoregulatory functions.

**Table 1:** TDDSs based on GA and proposed as potential candidates for the treatment of hepatocellular carcinoma.

Targeting Agents	Hybrid Materials	Therapeutic Agents	Therapeutic Approach	In Vitro Models	In Vivo Models	Ref.
GA	5-fluorouracil-GA conjugates	5-fluorouracil GA	Chemotherapy	BEL-7402 cells		[40]
GA	Furoxan-based derivatives of GA	NO donors GA	HCC chemotherapy	BEL-7402 and HepG2 cells		[41]
GA	Microshells of 18β-GA conjugated with tetramethylpyrazine and a small amino acid	GA Tetramethyl-pyrazine	HCC chemotherapy	HepG2 cells	HepG2 tumor-bearing BALB/c nude mice and H22 tumor-bearing Kunming mice	[43]
GA	Core-shell NPs made of PLGA coated with CS and loaded with GA	GA	HCC chemotherapy	HepG2 cells		[44]
GA	mPEG-PCL-PEI-GA copolymer NPs	Norcantharidin	HCC TDD	HepG2 cells	H22 tumor-bearing Kunming mice	[45]
GA	GA-decorated PEG-PLGA NPs	Artesunate	HCC TDD	HepG2, Hep3B and SMCC-7721 cells		[46]
GA	GA-modified HA NPs	Docetaxel	HCC TDD	HepG2 cells		[47]
GA	GA-modified HA NPs	Adenine	HCC TDD	HepG2 cells	HepG2 tumor-bearing female BALB/c nude mice	[48]
GA	Micelles based on GA-PEG-GA conjugates	Paclitaxel GA	HCC TDD	HepG2 cells		[49]
GA	PEG-Fmoc-GA micelles	Doxorubicin GA	HCC DD	HepG2 cells	HepG2 tumor-bearing female BALB/c nude mice	[50]
GA and derivatives	Liposomes based on GA derivatives linked with DSPE-PEG2000-NH2		HCC TDD	HepG2 cells	H22 tumor-bearing male BALB/c nude mice	[19]
GA	Liposomes based on soybean phospholipids, cholesterol, and 3-succinyl-30-stearyl GA	Wogonin	HCC TDD	HepG2 cells	HepG2 tumor-bearing BALB/c nude mice	[29]
GA	Liposomes based on egg phosphatidylcholine, cholesterol, and 3-succinyl-30-stearyl GA	Oxaliplatin	HCC TDD		Biodistribution in Kunming strain mice	[51]
GA	Cationic liposomes based on lecithin and complexes of GA and octadecylamine	Curcumin	HCC TDD	H22 cells	Intravenous and intratumoral injection in H22 tumor-bearing male Kunming mice	[52]
GA	GA-modified liposomes based on DSGPE-PEG2,000	Curcumin Combretastatin A4 phosphate	HCC TDD	Human HCC BEL7402 and mouse melanoma B16 cells	H22 tumor-bearing male BALB/c mice	[53]
GA galactose	Dual-ligand GA and galactose-modified CS NPs		HCC TDD	HepG2 and H22 cells	H22 tumor-bearing Kunming mice	[54]
GA galactose	GA and galactose dual ligand modified DSPE-PEG liposomes	Curcumin Capsaicin	HCC TDD	Cocultured HSCs and HepG2 cells	Subcutaneous H22 or H22+m-HSC tumor-bearing and orthotopic H22 tumor-bearing female BALB/c mice intravenous injection of H22 cells in female BALB/c mice	[55]
GA LA	Dual-ligand LA-CS-GA and CMCS-g-PA-based NPs	Doxorubicin	HCC TDD		Female Wistar rats intraperitoneally injected with *N*-nitrosodiethylamine and receiving a chloroform dose	[56]
GA LA	Dual-ligand LA-LMWH-GA based NPs	Doxorubicin	HCC TDD	HepG2 and HepG2/ADR cells		[57]
GA	GA-APS-disulfide bond-Cur nanomicelles encapsulated with RBCm	Curcumin	HCC TDD	HepG2 cells	NU/NU female nude mice inoculated with HepG2 cells	[58]
GA peanut agglutinin	GA and peanut agglutinin dual-ligand-modified liposomes based on soy lecithin and cholesterol	Doxorubicin	HCC TDD	MUC1-negative HepG2, MUC1-positive SMMC-7721 cells	SMMC-7721 tumor-bearing BALB/C-nude male mice	[59]
GA thiolated polymers	Thiolated CS and thiolated eudragit-based NPs reinforced with GA	5-fluorouracil	HCC TDD	HepG2 cells	Diethylnitrosamine and carbon tetrachloride-induced HCC in male Wistar Albino rats	[60]
GA HA	NPs based on HA-GA succinate conjugates	Doxorubicin	HCC-targeted and pH-responsive DD	HepG2 cells	HepG2 tumor-bearing BALB/c nude mice Sprague–Dawley rats for biodistribution study	[61]
GA HA	NPs based on HA modified with GA and l-histidine	Doxorubicin	HCC-targeted and pH-responsive DD	HepG2 cells	H22 tumor-bearing female BALB/c mice	[62]
GA	GA-PEG-HZ-PLA polymeric micelles		HCC-targeted and pH-responsive DD		H22 tumor-bearing Kunming mice	[63]
GA	NPs based on sHA-doxorubicin and HA-GA polymers	Capsaicin Doxorubicin	HCC-targeted and pH-responsive DD	Cocultured human HCC BEL-7402 and HSCs LX-2 cells pretreated with SP	Subcutaneous implantation of H22 or SP exposed-m-HSC/H22 cells, or of H22 cells for primary HCC, intravenous injection of H22 cells in female BALB/c mice	[64]
GA	sHA-doxorubicin/HA-GA micelles	Doxorubicin	HCC-targeted and pH-responsive DD	HepG2 cells	Human hepatoma PLC/PRF/5 cells implanted in BALB/c mice	[65]
GA	Micelles based on GA-PEG-PHIS-PLGA	Andrographolide	HCC-targeted and pH-responsive DD	Hep3B cells	HCC tumor-bearing BALB/c nude mice	[66]
GA	GA-functionalized mesoporous silica NPs presenting two cleavable bonds (an imine bond and an HZ group)	Doxorubicin Camptothecin	HCC-targeted and pH-responsive DD	HepG2 cells		[67]
GA	GA coupled to zirconium MOFs through 1,4-butanediamine chains	5-fluorouracil	HCC-targeted and pH-responsive DD	HepG2 cells	Biodistribution in Kunming mice	[68]
GA	Nanomaterial based on GA-functionalized GO	Doxorubicin	HCC-targeted and pH-responsive DD	HepG2 cells	HepG2 tumor-bearing BALB/c nude mice	[69]
GA	GA conjugated PPI dendrimers and multi-walled carbon nanotubes	Doxorubicin	HCC TDD	HepG2 cells		[70]
GA LA	Dual-ligand GA and LA-modified CS-based NPs	siPAK1	HCC-targeted delivery for gene therapy	Hep3B and HepG2 cells	Hep3B tumor-bearing female BALB/c nude mice	[71]
GA	NPs based on GA-CS-PEI using HBA as a linker to the drug	Bcl-2 siRNA Doxorubicin	HCC-targeted and pH-responsive DD for chemotherapy and gene therapy	HepG2 cells	HepG2 tumor-bearing BALB/c nude mice	[72]
GA HA	NPs composed of GA-HA and DSPE-PEG-PEI	Bcl-2 siRNA Doxorubicin	HCC-targeted DD for chemotherapy and gene therapy	HepG2 cells	H22 tumor-bearing female BALB/c mice	[73]
GA	PEI-GA NPs	Doxorubicin shAkt1	HCC TDD for chemotherapy and gene therapy	HepG2 cells	C57BL/6J mice inoculated with Hepa-1.6 cells	[74]
GA	LMWH PEI-GA conjugates	Plasmid DNA	HCC-targeted delivery for gene therapy	HepG2 cells	HepG2 tumor-bearing female BALB/c athymic mice	[75]
GA	GO-PAMAM-GA hybrids	Plasmid DNA	HCC-targeted delivery for gene therapy	Human SMMC-7721 cells		[76]
GA	Nanocomplex based on GA, PEG, PAMAM dendrimer and NGO conjugate	Anti-VEGFa siRNA	HCC-targeted delivery for gene therapy	HepG2 cells	HepG2 tumor-bearing NU/NU nude mice	[77]
GA	GA-TPP conjugate-based NPs		HCC PDT therapy	HeLa, HepG2 and K1 cells		[78]
GA	Derivative of SiPC with PEG, APDES, and GA		HCC targeted PDT therapy	HepG2 and Huh7 cells	HepG2 tumor-bearing male BALB/c nude mice	[79]
GA	Redox-responsive micelles based on PCL-SS-CMC-GA presenting a disulfide bond	Doxorubicin Pheophorbide A	HCC targeted NIR and redox-responsive DD	HepG2 cells	HepG2 tumor-bearing female BALB/c nude mice	[80]

APDES: 3-(ethoxydimethylsilyl)propylamine; APS: Angelica sinensis polysaccharide; CMC: carboxymethyl chitosan; CMCS-g-PA: carboxymethyl chitosan-g-polyacrylate; CS: chitosan; DD: drug delivery; DSGPE: 1,2-distearyl-sn-glycero-3-phosphoethanolamine; DSPE: distearoyl-phosphatidylethanolamine; Fmoc: 9-fluorenylmethyloxycarbonyl; GA: glycyrrhetinic acid; GO: graphene oxide; HA: hyaluronic acid; HBA: 4-hydrazinobenzoic acid; HCC: hepatocellular carcinoma; HZ: hydrazone; LA: lactobionic acid; LMWH: low molecular weight; MOFs: metal-organic frameworks; mPEG: polyethylene glycol methyl ether; MUC1: mucin 1; MWCNTs: multi-walled carbon nanotubes; NGO: nano-graphene oxide; NIR: near infrared; NO: nitric oxide; NPs: nanoparticles; PAMAM: poly(amidoamine); PCL: poly-ε-caprolactone; PDT: photodynamic therapy; PEI: polyethylenimine; PHIS: poly(l-histidine); PLA: polylactic acid; PLGA: poly(d,l-lactide-co-glycolide); PPI: polypropylene imine; RBCm: red blood cell membranes; siPAK1: shRNA targeting Akt1; SiPC: silicon phthalocyanine; siRNA: short interfering RNA; SP: substance P; HCC: hepatocellular carcinoma; SS: disulfide; TDD: targeted drug delivery; TPP: tetraphenylporphyrin.

## 4. GA-Functionalized Polymer-Based DDSs

Standard chemotherapy is centered on low-molecular-weight drugs, such as DOX, cisplatin, or gemcitabine, with a short half-life and off-target accumulation in healthy organs [81]. Due to unfavorable pharmacokinetics and a suboptimal biodistribution together with their unspecific mechanism of action and great volume of distribution, anticancer drugs induce severe side effects. 

Polymers are very large molecules containing many repeating subunits called monomers, and may be either synthetic (e.g., polyethylene glycol (PEG) or poly(d,l-lactide-co-glycolide) (PLGA)) or of natural origin (e.g., hyaluronic acid (HA) and chitosan (CS)). Homopolymers are chains of one type of monomer that are chemically linked, while copolymers are characterized by different units. Due to their high molecular weight, polymers have been classified as macromolecules since they present more than ten repeating units.

Polymer-based biomaterials have been considered very attractive DDSs able to improve drug solubility, cell permeability, and unspecific cytotoxicity and maintain the therapeutic drug concentration for an extended period within the organism. PLGA is one of the most successfully used biodegradable polymers for the development of nanoparticles (NPs) [82]. In the human body, PLGA NPs can escape from the vasculature through leaky endothelium, thus allowing their distribution at the tumor site due to the enhanced EPR [83]. Furthermore, PLGA is considered safe since it has good biocompatibility and no immunogenicity and is hydrolyzed to the non-toxic, biodegradable byproducts lactic acid and glycolic acid [84]. In particular, PLGA NPs coated with CS, thanks to their safety profile, good biocompatibility, and low levels of immunogenicity and toxicity, have been approved by regulatory agencies such as the US Food and Drug Administration (FDA) and the European Medicines Agency (EMA) as effective DDSs in humans [85]. An advantage of the CS backbone is that it can be modified by many functional groups. Toward this aim, Nocca and coworkers tested the in vitro cytotoxic effect of GA-loaded PLGA-CS NPs (2:1 polymer:drug ratio) on human HCC HepG2 cells [44]. NPs were able to transport a higher amount of GA inside the cells than simple diffusion through the cell membrane. However, not all of the GA carried by the NPs was immediately bioavailable since about 5% (after 2 h of incubation) of the GA was released into the cytoplasm. However, even if GA (500 µM) was unable to reach the cellular compartments in the encapsulated form with the same efficacy as the free form, its concentration was high enough to affect cellular viability to the same extent as free GA [44].

Similarly, GA-decorated polymeric NPs loaded with norcantharidin (NCTD), a chemically synthesized and FDA-approved drug for cancer treatment [45], were tested for active liver targeting. The copolymer methoxy poly(ethylene glycol)-poly(ε-caprolactone) (mPEG-PCL, MEP) contains a core for the attachment of hydrophobic drugs and a hydrophilic stabilizing surface and is known for its biodegradation effectiveness and sustained drug release properties. Zhang and coworkers [45] prepared a tri-block mPEG-PCL-PEI (MPP) copolymer by also introducing a polyethylenimine (PEI) block which acts as a “bridge” to link MEP and GA. Data confirmed that these GA-NPs not only overcome the poor solubility of NCTD but also enhance the tumor-targeting effect, ultimately improving the therapeutic efficacy against HCC. The in vitro cytotoxicity assay showed that GA-NCTD NPs exhibited a better antitumor effect when compared to NCTD NPs or free NCTD, indicating that the drug was taken up more effectively by HepG2 cells. Apoptosis-inducing effects and cell cycle arrest in S and G2 phases were also higher for GA-NCTD NPs when compared to other groups. In vivo evidence confirmed the antitumor potential of GA-NCTD NPs using H22 tumor-bearing mice. Although all the tested NPs containing NCTD provided efficacy in preventing tumor growth compared to unloaded NPs, mice treated with GA-NCTD NPs exhibited the strongest inhibitory efficacy with a median survival time of 68 days compared to 56 days in the NCTD NPs group. Additionally, xenograft tumor tissues treated with GA-NCTD NPs displayed lower prognostic marker levels of Ki-67 and microvessel density compared to other groups, indicating a significant suppression of tumor proliferation and angiogenesis, respectively.

Similarly, Pan and coworkers tested GA-targeted PEG-PLGA NPs to improve the therapeutic efficiency of artesunate (ART) in the treatment of HCC. ART is an antimalarial drug extracted from the traditional herb *Artemisia annua* with anticancer activity [46]. Data confirmed increased GA-ART NPs cytotoxicity on HepG2, Hep3B and SMCC-7721 human HCC cell lines compared with free ART. Interestingly, the addition of GA into the medium reduced the cytotoxicity of GA-ART NPs, supporting that GA might competitively bind to cell surface receptors that are essential for GA-targeted DSs entering into HCC cells.

HA, a naturally occurring hydrophilic acid mucopolysaccharide containing repeating *N*-acetyl-d-glucosamine and d-glucuronic disaccharides, displays exceptional properties such as biocompatibility, biodegradability, and low toxicity and represents an ideal carrier polymer to produce NPs for the targeted delivery of drugs. In addition, HA can actively target the surfaces of cancer cells, such as HCC cells, by binding the CD44 receptor. Xue and coworkers tested in vitro GA-modified HA NPs as carriers for the model drug docetaxel (DTX) [47]. DTX is known to bind the β-tubulin subunit of microtubulin and to induce microtubule stabilization and prevent its de-polymerization [86] but is also able to enhance the polymerization of cellular tubulin by binding α-tubulin. Confocal laser scanning microscopy analysis confirmed the cellular uptake efficiency of GA-HA NPs in HepG2 cells compared to MCF-7 breast cancer cells that typically do not express GA receptors. A slight drug uptake observed in MCF-7 cells may have been due to the binding affinity of HA to its receptor CD44. Additionally, as demonstrated by others, GA coincubation with DTX/GA-HA NPs inhibited the uptake of NPs through competition with cellular GA receptors. Compared with DTX alone, DTX/GA-HA NPs strongly reduced HepG2 cell proliferation and induced apoptosis. At a molecular level, free DTX-exposed cells showed partial tubulin polymerization and a slight change in cell morphology, while DTX/GA-HA NP exposure improved tubulin polymerization around nuclei and reduced cytoplasmic tubulin with markedly distorted cellular morphology.

Using the same DDS, Wu and coworkers [48] tried to target liver cancer cells with adenine (ADE), an anticancer agent able to induce cell arrest in the S phase in human hepatic carcinoma cells, leading to tumor cell proliferation arrest and subsequent cell apoptosis. Interestingly, data reported that GA-HA NPs were more efficient in targeting liver cancer cells than normal liver cells (due to the higher presence of GA receptors in tumor cells than in normal cells), leading the authors to hypothesize reduced drug side effects on normal tissues. However, the effects obtained in vitro on HepG2 cells were also confirmed in vivo in Kunming mice using the fluorescent tracer 3′-tetramethylindotricarbocyanine iodide (DiR). Real-time in vivo images showed that GA-HA NPs increased DiR accumulation in liver tissues compared with free DiR groups. Drug selective targeting was also confirmed since the ADE concentration in liver tissue for the ADE/GA-HA NPs group was 3.7-fold higher than in the free ADE group with a prolonged elimination rate from blood circulation. In vivo evidence for the antitumor effect of ADE/GA-HA NPs was also demonstrated by a reduced tumor volume and weight in the ADE/GA-HA NPs group compared to the GA-HA NPs and free ADE groups in a HepG2 xenograft BALB/c nude mice model. At the molecular level, ADE/GA-HA NPs reduced the expression of proliferating cell nuclear antigen (PCNA), an immunohistological marker for the in vivo evaluation of tumor growth, and increased apoptotic DNA fragmentation (TUNEL assay) when compared to other groups.

Amphiphilic copolymers may be used to produce micellar DDSs. In particular, one has to note that the micelle surface can be modified with GA to target cancer cells. Furthermore, micellar DDSs can be taken into consideration as a promising strategy for multi-drug delivery. In fact, the development of combination therapies is essential to achieve synergic therapeutic effects by simultaneously affecting multiple pathways involved in cancer promotion and progression and by reducing toxicity and side effects. Wu and coworkers [49] used triblock GA-PEG-GA-based self-assembled micelles loaded with PTX to selectively deliver drugs to hepatic cells thanks to the targeting properties of GA, as studied by an in vitro cytotoxicity evaluation on HepG2 cells and by an in vivo biodistribution investigation in rats.

More recently, Yang and coworker [50] tested a novel polymeric micellar carrier based on PEG-derivatized GA for the co-delivery of GA and DOX as a combined anti-cancer treatment. In addition, a drug-interactive motif 9-fluorenylmethoxycarbonyl (Fmoc) was incorporated into the system to enhance the drug loading capacity and improve the stability of the drug-loaded emulsion and micellar formulation [87]. In xenograft HepG2 tumor-bearing mice, DOX distribution from DOX/PEG-Fmoc-GA micelles 24 h after administration showed higher DOX accumulation in tumors when compared to free DOX. Interestingly, the authors observed a lower DOX accumulation in the heart, supporting less severe cardiotoxicity side effects from the DOX with PEG-Fmoc-GA micelle carriers.

## 5. GA-Functionalized Liposomes

An innovative kind of DDS is represented by a combination of lipids and amphiphilic polymers. Liposomes (Lip), spherical bilayer vesicles formed spontaneously with phospholipids dispersed in water, are well known for their application as DDSs, and their surface may be modified by GA to target liver cancer cells. However, liposomes have critical problems such as drug leakage during storage, burst drug release, normal tissue toxicity, and immune response, so their performance may be improved by using polymers, which are more stable than lipid molecules and less prone to undergo oxidative degradation phenomena [88,89]. A very common way to coat liposomes with polymers is to covalently bind polymers to the polar head of phospholipids. Decorating a lipidic vesicle with a hydrophilic polymer such as PEG can improve its physicochemical stability, stealth properties, and mucopenetrating capability while giving it the possibility to introduce functions suitable for targeting purposes [89]. Sun and coworkers tested distearoyl-phosphatidylethanolamine (DSPE)-PEGylated liposomes functionalized with GA in order to evaluate cellular location in vitro and tumor targeting in vivo [19]. Interestingly, this study evaluated the effects of different GA isomers (the *trans* form 18α- and the *cis* form 18β-GA) as well as of other derivatives obtained by the removal of 11-carbonyl in the ring structure of GA (11-deoxy-18β-GA) or the removal of the hydroxyl at C3 through C3-acetylation (3-Ace-GA). In particular, 11-deoxy-18β-GA does not induce a pseudoaldosterone effect and possesses better anti-inflammatory, antiulcer, and antiallergic activities [19]. In comparison with long-circulation liposomes, 18β-GA-Lip and 3-Ace-GA-Lip were found to be more accumulated around HepG2 cells in a short time and were transferred into HCC tumors in vivo in H22 tumor-bearing nude mice for a longer time.

Some papers reported the targeting properties of 3-succinyl-30-stearyl GA using conventional liposomes based on phospholipids and cholesterol in order to deliver wogonin (WG) [29] or oxaliplatin (OX) [51]. These findings demonstrated that GA-modified liposomes can produce a better drug delivery to the liver, as demonstrated by in vivo experimental models employing HepG2 tumor-bearing BALB/c nude mice for WG-loaded liposomes and Kunming strain mice for OX-loaded liposomes, without severe toxicity signs. 

Similarly, Chang and coworkers [52,90] tested GA-targeting properties using conventional liposomes carrying a positive charge. Cationic liposomes, which are largely studied for gene delivery, have been tested during this decade since it is known that tumor cells possess high concentrations of negatively charged glycoproteins on their exterior surface [91], leading to increased local retention of this preparation in the tumor tissue and reduced distribution to other tissues. However, since these liposomes themselves can produce toxicity to normal tissues and elicit an immune response, an efficient method of systemic delivery of liposomes to tumor tissues is required to enhance the localization of payloads. GA-modified octadecylamine-charged liposomes were loaded with curcumin (CUR) (GA-CUR-Lip). CUR is known for its anticancer activity because it blocks cell NF-κB signaling. GA-CUR-Lip were tested in vitro, and it was demonstrated that, compared to free CUR, GA-CUR-Lip strongly promoted apoptosis of hepatocellular cancer H22 and HepG-2 cells and enhanced the inhibition of cancer cell proliferation [90]. Furthermore, they may be efficiently used in vivo through intravenous and intratumoral injection. One has to mention that the locoregional delivery of liposomal agents by intratumoral injection can help to eliminate the anatomic and physiological barriers present in the tumor mass. In particular, in vivo investigations via intratumoral injection in H22 xenograft Kunming mice [90] demonstrated that GA-CUR-Lip, compared to free CUR, also exhibited a much better capability to inhibit tumor growth, inducing apoptosis of tumor tissue, reducing VEGF expression, and upregulating caspase-3 in tumor tissues. In addition, the results showed that unlike CUR alone, whose antitumor efficacy was much weaker than that of adriamycin (a first-line chemotherapy drug), high-dose GA-CUR-Lip showed efficacy similar to adriamycin. These results revealed that cationic liposomes could be used to encapsulate drugs such as curcumin, and the modification of cationic liposomes with GA might shade the positive charge, which could overcome their limitations and improve the stability in serum.

Recently, liposomes provided a promising chance to overcome the problem of an effective codelivery of multiple drugs, since, as said before, chronic administration of a single drug is often associated with undesirable side effects or drug resistance, which may cause failure in cancer therapy. This approach was examined by Jiang and coworkers [53], who evaluated GA-modified PEGylated liposomes in order to increase active targeting to HCC cells and tissue of CUR and combretastatin A4 phosphate (CA4P). CA4P is known to act at the level of tumor vasculature, inducing vasoconstriction and death of neoplastic cells due to insufficient blood supply. The introduction of GA in the DDS increased cellular uptake of drug-loaded liposomes, leading to increased accumulation of drugs in tumor cells and higher cytotoxicity in human HCC BEL7402 cells. The in vivo efficacy of CUR-CA4P/GA Lip in H22 tumor-bearing mice showed an inhibition rate of CUR-CA4P/GA Lip higher than that induced by CUR-CA4P Lip, confirming the existence of an active liver-targeted delivery.

## 6. Dual-Ligand TDDSs

**Figure 3:**
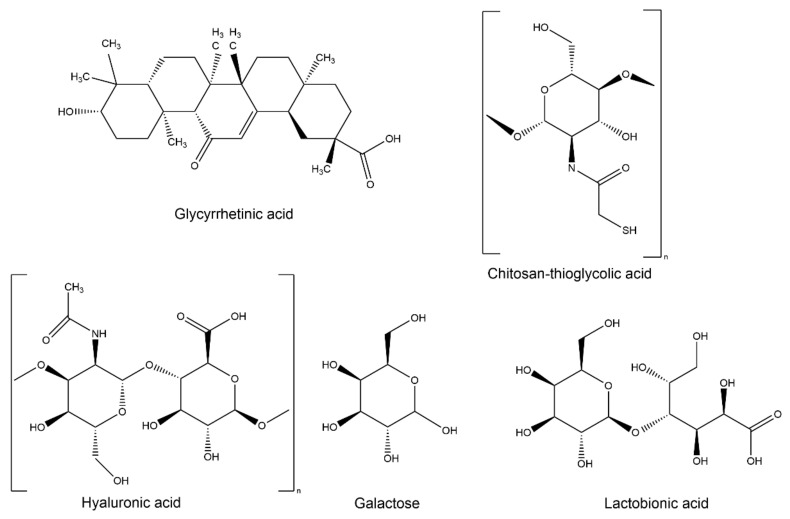
Examples of molecules used as ligands for the functionalization of DDSs and their targeting to HCC cells. DDSs: drug delivery systems; HCC: hepatocellular carcinoma.

Due to the limited quantity of receptors on the target cell membrane, the cellular uptake of single-ligand modified NPs is restricted. Even if the dose increases, at a certain point, the response becomes saturated, the cellular uptake will not increase, and all biological responses reach a maximum [71]. To overcome this limitation and improve the cellular uptake efficacy, dual-ligand TDDSs have been developed with the aim of reinforcing the tumor-targeting specificity (Figure 3).

Endogenous lectins are present on many normal and cancer cells and participate in several biological functions, working as receptors and mediating endocytosis of specific glycoconjugates. Carbohydrate-specific receptors present in the cell membrane, able to recognize carbohydrate epitopes, play an important role in cellular adhesion and recognition processes, distinguishing specific complex oligosaccharides with an elevated affinity and avidity. Recent research studies have shown that ASGP-R is a special lectin expressed on the cell membrane of liver cells and overexpressed in several human tumoral hepatocytes [92]; it can internalize molecules exposing the carbohydrate residue galactosamine through clathrin-type receptor-mediated endocytosis [93]. Many molecules containing an ASGP-R binding site have been considered as potential ligands of interest for the hepatic delivery of therapeutic agents.

Li and coworkers [68] confirmed improved liver targeting of dual GA and galactose (Gal)-modified CS NPs as a novel targeting vehicle for HCC with no apparent systemic side effects in Sprague–Dawley rats.

An innovative dual ligand-modified TDDS was recently proposed by Qi et al., who developed GA and Gal-functionalized DSPE-PEG liposomes for the co-delivery of CUR and capsaicin (CAPS), known for its anticarcinogenic, antiproliferative, and antioxidative effects [94], for liver cancer treatment [55]. The drugs loaded in this system are targeted to different components of the tumor environment. Liver-specific pericytes, known as HSCs, are located in the perisinusoidal space of the liver and are fundamental for the development of HCC. Under normal conditions, HSCs exist in a quiescent state, but in the liver tumor microenvironment, HSCs are activated (aHSCs), transitioning to a myofibroblast phenotype with proliferative, migratory, and invasive capabilities, representing one of the driving factors for tumor progression and drug resistance [95,96]. CAPS, a natural product of capsicum species, can induce significant antifibrosis effects by inhibiting the proliferation of HSCs and might reduce drug resistance and metastasis of tumor cells by blocking the activation of HSCs in the tumor microenvironment through targeting the TGF-β-Smad signal pathway [97]. The in vitro cellular uptake of GA- and Gal-Lip were determined in HepG2 cells using an FITC tracer. Compared to the FITC-Lip treatment, the cells treated with Gal- or GA-modified Lip showed stronger fluorescence, while the FITC-GA-Gal-Lip group showed a greater fluorescent intensity than a single ligand, suggesting that the introduction of Gal and GA promoted cellular uptake through the Gal receptor- and GA receptor, respectively. The anticancer properties of CUR and CAPS loaded in Gal-GA-modified Lip were demonstrated in a HSCs+HepG2 model, which mimics the tumor microenvironment. In vivo targeting was also confirmed in a new subcutaneously implanted B16/H22 cells-bearing mice model, in which mouse melanoma B16 cells do not express GA-R and ASGP-R receptors. Using DiR as a tracer, the authors demonstrated no significant differences between DiR/Lip and DiR/GA&Gal-Lip in the B16 tumor, while a greater fluorescent intensity and longer-lasting time in the H22 tumor region was observed in the DiR/GA&Gal-Lip group than in the DiR/Lip group. In subcutaneously implanted H22 tumor-bearing mice, in comparison with free CUR+CAPS, the coloaded Lip exhibited a higher antitumor effect, with a tumor inhibition rate of 94.5% for CAPS-CUR/GA&Gal-Lip than that of CAPS-CUR/Gal-Lip (82.5%). The same effects were also observed in orthotopic implanted H22 tumor-bearing mice, a model that better mimics actual liver cancer development due to stromal cells and complex cellular factors in the liver. More interestingly, in female BALB/c mice injected with murine H22 cells and HSCs, CAPS-CUR/GA&Gal-Lip showed a tumor inhibition rate of 88.41%, demonstrating that the dual-ligand-modified Lip has better inhibition of tumor development when compared to other formulations. Finally, intravenous injection of H22 cells produced lung metastases that were better reduced by CAPS-CUR/GA&Gal-Lip when compared to the other groups. 

Lactobionic acid (LA), containing gluconic acid and a Gal moiety, has rapidly emerged as an alternative targeting molecule thanks to the selective cellular uptake capacity of hepatoma-targeting chemotherapy due to its ability to bind ASGP-R [98]. Hefnawy and coworkers [56] tested the effects of DOX-loaded GA-LA dual ligand-decorated CS NPs, prepared through electrostatic interactions between the positively charged DOX and the negatively charged CS derivatives, as a liver-targeted delivery system. In vitro HepG2 cellular uptake demonstrated that although the GA-ligated NPs did not show significant enhancement in cellular uptake, the presence of GA ligand with the LA moieties resulted in an improved cellular uptake with a synergistic effect. In vivo anticancer activity was assessed in a liver cancer model in Wistar rats injected with *N*-nitrosodiethylamine receiving a chloroform dose. The drug-loaded NPs were able to significantly improve the levels of various serum biomarkers by reducing circulating α-fetoprotein and transaminases. In addition, compared to the free drug, dual-ligands CS NPs showed higher therapeutic efficiency as indicated by their ability to induce apoptosis rather than necrosis. These NPs also had the advantage of allowing regeneration of the tissues and restoring the normal liver structure, as well as also possessing a better safety profile compared to the conventional DOX. Interestingly, an effect was also observed for dual-ligand CS NPs without DOX, probably due to the hepatoprotective and anticancer effects of the two attached ligands as well as CS.

However, the introduction of a second ligand molecule in the DDS, besides GA, can lead to effects opposite to those desired. Du and coworkers tested GA-LA dual-ligand properties of DOX-loaded NPs, prepared from a low-molecular-weight heparin (LMWH)-derived polymer [57]. In this study, self-assembled LMWH-GA NPs were coupled to LA to develop a dual ligand drug carrier (LA-LMWH-GA). Data demonstrated that the hydrophilic LA interferes with the self-assembly of the nanostructures, allowing higher encapsulated drug release. In vivo data revealed reduced DOX blood levels for LA-coupled NPs. The cellular uptake of DOX-loaded NPs in adriamycin-resistant HepG2 (HepG2/ADR) cells showed higher cellular uptake compared to free DOX; this effect may be attributed to the GA-mediated NP uptake since competition with free GA was observed. Additionally, the addition of free LA reduced LA-LMWH-GA uptake, demonstrating that both GA-mediated and LA-mediated endocytosis were involved. However, DOX/LA-LMWH-GA showed reduced cellular uptake compared to DOX/LMWH-GA, supporting that the two endocytosis mechanisms can reciprocally interfere with their internalization. This effect can also explain the higher cytotoxicity and enhanced cell apoptosis of DOX/LMWH-GA compared to the dual-modified NPs. A hypothesis is that when LA is conjugated, the decreased cellular uptake efficiency of NPs can be due to the reconstruction of hydrophobic/hydrophilic balance in the NPs, with minor hydrophobic GA groups on the NP surfaces that in turn reduce GA-mediated endocytosis. In conclusion, the authors suggest that LA modification on LMWH-GA could decrease the uptake of DOX by weakening the effect of micropinocytosis and caveolae-mediated endocytosis in cell internalization.

Since there is an increased interest in the research of materials for targeted delivery, a new DDS has been developed by producing nanomicelles from *Angelica sinensis* polysaccharides (APS), the main active components of this plant [58]. APSs represent a valid alternative to classical natural polysaccharides for drug delivery carriers, such as CS, alginate, heparin, and HA, since APSs have liver-targeting properties, such as targeting the mannose receptor and the GAL receptor on the surface of HCC cells. APS nanomicelles were further functionalized with GA in order to improve liver targeting to deliver CUR to hepatoma carcinoma sites and encapsulated with red blood cell membranes (RBCm) to escape from immune system clearance. APS micelles showed higher toxicity to HepG2 cells compared to classical HA micelles, while the latter have selective toxicity for human breast adenocarcinoma MDA-MB-231 cells. These results indicated the better HepG2 targeting and delivering ability of APS micelles than HA micelles, while the high expression of CD44 receptor in MDA-MB-231 cells resulted in better targeting properties of HA micelles. In addition, after coating micelles with RBCm, the cytotoxicity of GA-APS-CUR@RBCm was only a little higher than uncoated micelles. Antitumor growth efficacy was evaluated in nude mice bearing HepG2 cancer cells, demonstrating that the RBCm-coated and uncoated GA-APS-CUR had the best inhibition effects. These findings indicate that GA-APS micelles can be used for targeting liver tumor tissue and improving anticancer activity, increasing drug concentration in the tumor sites. In addition, GA-APS-CUR@RBCm improved the number of splenic CD4+ and CD8+ T cells and the serum levels of cytokines better than uncoated and HA micelles, demonstrating the activation of antitumor immunity with promising effects for the treatment of HCC.

Using a similar approach, GA and peanut agglutinin (PNA), a plant lectin protein able to bind to β-d-galactosyl-(1–3)-*N*-acetyl-d-galactosamine (Gal-β(1–3) GalNA), were used to prepare dual-ligand-modified DOX-loaded liposomes (DOX/GA-PNA-Lip) to improve liver targeting and anticancer efficacy [59]. Gal-β(1–3) GalNA is a core structure of mucin 1 (MUC1), a mucin associated with different types of cancer cells and abnormally overexpressed in about 80% of epithelial cancer cells. DOX/GA-PNA-Lip significantly increased the cellular uptake of DOX in hepatocellular carcinoma SMMC-7721cells compared to other single-ligand liposomal formulations via caveolae-mediated endocytosis and micropinocytosis mediated by GA receptors and MUC1 for GA and PNA, respectively. In vivo data confirmed that this dual-ligand DDS had the highest inhibitory effects on tumors when compared to a single-ligand group in a SMMC-7721 cell xenograft model in BALB/C-nu mice, alleviating the systemic toxicity of DOX. 

The membrane is able to internalize compounds through several specialized mechanisms and, among these, cell surface thiol groups (-SH) can be used to improve cellular internalization of materials with thiol-reactive groups [99]. Thanks to the sulphur-containing, amino acid-rich subdomains of glycoproteins normally present in biological systems like cancer cells, thiolated polymers can be potentially useful for targeted drug delivery [100]. In particular, the evidence that some cancer cells are reported to express a higher level of exofacial thiol suggests the potential employment of thiolated polymers for DDSs targeted to cancer cells. Bhat and coworkers [60] tested new nanocomposites obtained from thiolated CS and eudragit (a pH-dependent soluble polymer used in the design of enteric-coated formulations and as a carrier) with reinforcements of GA and loaded with 5-FU to selectively target HCC. In vivo data using diethylnitrosamine- and carbon tetrachloride-induced HCCs in rats showed that serum transaminases, alkaline phosphatase, γ-glutamyltransferase, and total bilirubin levels were significantly reduced compared to controls. HPLC analysis of the liver cell homogenates revealed the presence of 5-FU at higher concentrations in animals treated with the drug-loaded nanocomposites that in those treated with 5-FU alone. The potential mechanism was investigated by in silico analysis showing that both GA and thiolated eudragit exhibited strong binding affinities with the active site of the liver receptor homolog 1 (LRH-1). LRH-1 up-regulates cyclins D1/E1 and c-Myc genes and induces proliferation and tumor growth. LRH-1 consists of a large hydrophobic pocket within the ligand-binding domain based on endogenous ligand binding, with this feature being fundamental for the development of new ligands able to modulate its activity. Thus, due to their hydrophobic properties, GA and thiolated eudragit could specifically inhibit LRH-1, reducing cell migration, invasion, and sphere formation.

## 7. Environment-Responsive GA-Functionalized DDSs

**Figure 4:**
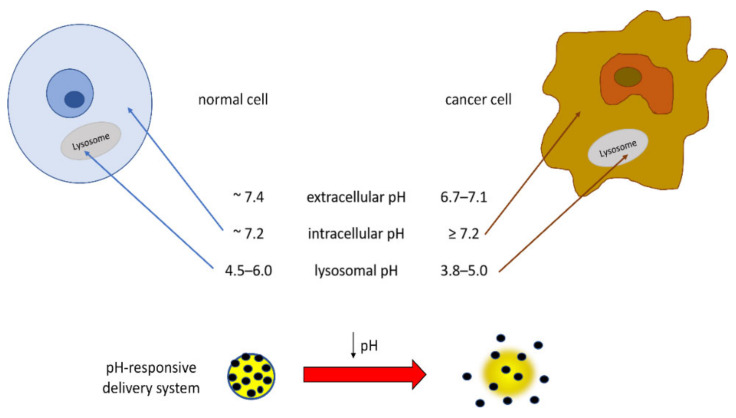
pH-responsive drug delivery systems represent an innovative approach in cancer therapy, taking advantage of the slightly acidic extracellular pH environment of solid tumors as well as of acidic lysosome pH, and thus allowing a more efficient drug release at the tumor level.

Of particular interest in the field of TDDSs is the development of materials that respond to the acidic pH of tumor extracellular tissues and intracellular organelles such as endosomes and lysosomes. In fact, the dysregulation of cellular pH, closely connected to hypoxic conditions, is common in solid tumors, and this tumoral acidic microenvironment can promote migration, invasion, and metastasis through various mechanisms [101]. The pH value of normal tissues and the blood environment is neutral (~7.4), whereas due to increased proton production and poor proton clearance, the pH of solid tumor tissues is slightly acidic (6.7–7.1). Intracellular pH is reported to be ~7.2 in normal cells and ≥7.4 in cancer cells [102]. Additionally, the lysosomes and intracellular compartments of tumor cells are more acidic than those of normal cells, very likely in relation to the presence of H^+^-ATPases, such that the lysosomal pH value in cancer cells was reported to be in the range low as 3.8–5.0 for cancer cells and in the range 4.5–6.0 for normal cells [103,104,105]. These pH differences can be applied for preparing DDSs releasing the drug at the target site, thus reducing the side effects of anticancer drugs and also improving the effectiveness of the chemotherapy (Figure 4). In order to improve the bioavailability of the drug, delivery systems responsive to environmental stimuli such as pH have been pursued.

CS and HA show pH-responsive properties via swelling or shrinking in the external media, producing valuable DDSs able to induce a burst release by a modification in the pH.

DOX-loaded HA-GA succinate (HSG) conjugate-based NPs (HSG/DOX NPs) were tested for their liver-targeting and pH-sensitive properties [61]. Both HA and GA can have a role in targeting liver tumor cells through CD44 receptors and GA receptors, respectively. In this DDS, GA succinate was conjugated to HA via its hydroxyl group and not via the carboxyl groups since the carboxyl groups are the natural HA recognition sites. Data showed that HSG/DOX released approximately half of DOX in phosphate-buffered saline (PBS) at pH 5.5 after 48 h, which was higher at the other pH tested (6.5 and 7.4) [61]. This can be due to the pH-dependent solubility of DOX, with a slow release of DOX in the bloodstream (pH 7.4) able to extend drug circulation time, and with a faster release at lower pH (5.5–6.5). In addition, HSG/DOX NPs exhibited a GA concentration-dependent cytotoxicity in HepG2 cells, reaching a plateau at a higher GA ratio due to saturation mechanisms. In vivo data on Sprague–Dawley rats demonstrated that liver targeting capacity was GA ratio-dependent since the increased density of GA on the surface of the NPs resulted in higher binding affinity to the liver region by GA receptor-mediated endocytosis. Additionally, in vivo analysis in HepG2-bearing BALB/c nude mice confirmed that the NPs synthesized by modifying the HA hydroxyl groups not only had better liver-targeting properties but also presented higher tumor-targeting efficiency compared with NPs synthesized by modifying carboxyl groups.

Tian and coworkers [62] prepared a novel DOX/GA-HA-Histidine (GHH) DDS for the dual function of liver-targeted delivery via GA receptor-mediated endocytosis and pH-responsive drug release at the level of lysosomes through protonation of the imidazole group of histidine (His). Data confirmed better in vitro DOX release under an extracellular tumoral condition (29.8% at pH 6.8) when compared to physiological conditions (21.4% at pH 7.4). However, at an intralysosomal pH of 5.0, the DOX release rate was much faster, with 58.9% of DOX released after 24 h. In fact, under physiological conditions (pH 7.4), the NPs have a stable GA-His hydrophobic core, slowly releasing DOX. At pH 6.8, DOX is released faster due to the partial protonation of the His imidazole ring and slight NP swelling. At the intralysosomal pH (5.0), the main parts of the imidazole rings are protonated, repelling each other and moving out of the hydrophobic core, causing evident NP swelling. The cellular uptake of DOX from the GHH NPs, analyzed with the autofluorescence of DOX in HepG2 cells, indicated more DOX from the GHH NPs in the cytoplasm and nucleus compared to HA-GA NPs. The in vivo anti-hepatoma efficacy in H22 tumor-bearing mice confirmed the pH-responsive properties of this preparation since the inhibition efficiency of DOX/GHH NPs was higher than that of the other DOX treatment groups. 

Recently, pH-sensitive polymeric micelles modified with a hydrazone bond have been considered a promising tool for effective cancer therapy. Hydrazone bonds, formed by the carbonyl group with hydrazine, have an acid-sensitive chemical structure; they are stable under physiological pH conditions but are rapidly hydrolyzed in the slightly acidic microenvironment of hepatoma cells. Zheng and coworkers tested the pharmacokinetics of GA liver-targeted delivery using a pH-sensitive polymeric DDS based on PEG-PLA hydrazone bond-modified micelles (GA-PEG-HZ-PLA) [63] loaded with coumarin-6 as a fluorescent tracer. The micelles have shown in vivo long-circulation properties and relative intake and targeted efficiency since GA-PEG-HZ-PLA were more successfully accumulated in the liver and tumor in H22 tumor-bearing mice when compared to PEG-PLA and PEG-HZ-PLA.

An intelligent pH-sensitive DDS of CAPS/GA-sulfated HA (sHA) DOX-loaded NPs was developed for liver-targeting co-delivery of DOX and CAPS [64]. The potential role of CAPS against tumor growth has been described above [55]. Furthermore, CD44 receptors are specifically overexpressed on aHSCs, so that HA-based NPs can specifically target these cells. On the other hand, HA polymers can be degraded by hyaluronidase forming low-molecular-weight fragments, which could promote tumor proliferation and migration; sulfation to the -OH groups of HA polymers (to form sHA) can block enzymatic HA degradation and prevent dangerous effects of its products. Both DOX and CAPS showed pH-responsive release behaviors from the NPs at pH 6.8, while the acid-liable hydrazone bonds between sHA and DOX were significantly broken at pH 5.5, resulting in the release of large amounts of drugs. Significantly, CAPS/GA-sHA-DOX NPs showed higher cytotoxicity and minimum migration rate than other preparations in HCC BEL-7402 cells due to increased drug uptake via GA-R-mediated endocytosis. In vitro experiments were based on a co-culture of human-derived HSCs (LX-2) and HCC BEL-7402 cells exposed to substance P (SP). In fact, SP, a neurotransmitter able to regulate the tumor microenvironment via the neurokinin-1 receptor (NK-1R), is supposed to activate normal HSCs to aHSCs through the SP/NK-1R signal pathway [106], while CAPS is mentioned as an SP inhibitor [107]. Furthermore, one of the in vivo models of female BALB/c mice employed in this study is based on the subcutaneous implantation of SP-exposed, mouse-derived HSCs and H22 cells. Altogether, the findings show that CAPS/GA-sHA-DOX NPs can inhibit the activation of HSCs, decrease drug resistance and metastasis of HCC cells by cutting off the cross-talk between HSCs and HCC cells, and promote cellular drug uptake by aHSCs and HCC cells through CD44 and GA receptors, respectively [64].

Additionally, Li et al. used sHA to develop a DDS based on sHA-DOX/HA-GA micelles, which showed HCC-targeted and pH-responsive properties both in vitro against HepG2 cells and in vivo in H22 cell-bearing BALB/c mice [65].

Another excellent candidate polymer for pH-sensitive drug release is poly(l-histidine) (PHIS) due to the protonation of its imidazole groups in acidic cytoplasm (pH < 6.5), other than its ability to escape from the endosome. Using GA and PHIS conjugated with PEG-PLGA (GA-PEG-PHIS-PLGA, GA-PPP), the liver-targeting properties of andrographolide (AGP)-loaded micelles were tested [66]. The AGP/GA-PPP micelles exhibited stronger antitumor efficiency in HCC tumor-bearing BALB/c nude mice than free AGP and AGP loaded in non-functionalized micelles. In fact, tumor volume and weight were smaller than those in other treated groups, supporting the fast drug release and efficient endosomal escape of these pH-sensitive micelles. Additionally, AGP/GA-PPP showed no overall toxicity compared to the other treatments.

Similarly, a pH-triggered mesoporous silica nanoparticle (MSN)-based nano-vehicle for the dual delivery of DOX and camptothecin-PEG (CPT-PEG) has been prepared and decorated with GA (CPT-PEG@MSN-DOX-GA) to target HepG2 cells [67]. The combination of DOX and CPT is of particular interest due to their strong capability to inhibit topoisomerases. The highly insoluble CPT was derivatized with a cleavable disulfide PEG chain to improve its loading within the MSN and the thiol redox-sensitive drug release; in fact, glutathione cleavage of the CPT prodrug releases free CPT, exerting a synergistic effect in combination with DOX. This system contains two cleavable bonds: an imine bond (through which GA is bonded to the NP) at its outer part, which may be cleaved at a slightly acidic pH (6.8), and a hydrazone group (used to link PEG to the surface of MSN) that may be cleaved at a more acidic pH (4.5). GA-decorated DDSs were selectively internalized into HepG2 by receptor-mediated or electrostatic interaction-mediated endocytosis and showed great cytotoxicity towards HepG2 cells. DOX released from CPT-PEG@MSN-DOX-GA was rapidly localized in the nuclei, very likely due to an efficient endosomal escape related to the anionic GA charge. 

Metal-organic frameworks (MOFs) are a new generation of materials consisting of metal ion clusters linked by multitopic organic ligands, forming extended network structures. Due to their capability to be used as DDSs, there is an increased interest in the development of nano MOFs (NMOFs). MOF materials are considered good candidates for small-molecule drug delivery thanks to their good biological safety and porous nature, which is responsible for their high drug loading and long drug release time. In particular, zirconium MOFs exhibit high stability and low toxicity [68]. In the system developed by Li et al., GA was attached to MOFs designed with zirconium as the metal ion center (GA-MOFs) to improve tumor recognition; this system was employed for 5-FU delivery to liver cancer cells. Overall, 5-FU@GA-MOFs led to a high drug release efficiency at low pH values and exhibited a pH-dependent release pattern, probably due to the partial collapse of the crystalline structure of MOFs at low pH. In vivo experiments on KM mice showed that 5-FU@GA-MOFs prolonged the blood circulation time, enhanced the liver targeting efficiency, and reduced the cardiotoxicity and nephrotoxicity of 5-FU.

A pH-sensitive DDS based on GA-functionalized graphene oxide (GA-GO) was prepared by Zhang and coworkers and used as an effective nanocarrier for the targeted delivery of DOX into liver cancer cells [69]. In addition, GA was used as a ligand to target liver cells and target mitochondria. As said before, mitochondria-mediated apoptosis (MMA) is an innovative approach for cancer therapy due to the presence of cell-suicide factors in mitochondria; unfortunately, mitochondria are highly impermeable organelles, and their low permeability represents a strong limit for TDD. GA increases mitochondrial permeability, opening transition pores through hydrogen peroxide production, which in turn oxidizes thiol groups and endogenous pyridine nucleotides [34]. The DOX release from this GA-GO@DOX system is affected by the hydrogen bonding and π-π interactions between DOX and GO, which is also modulated by pH value. In particular, at low pH values, typical in the tumor environment and lysosomes, the interaction is weak, resulting in a high release rate of DOX [69]. Under neutral conditions, such as in blood plasma and normal tissues, the GA-GO@DOX reduces the systemic distribution of DOX while inducing fast DOX release at pH 5.5 and pH 6.6. In vitro data showed GA-GO uptake in HepG2, and this uptake was reduced through competitive transport of free GA. Additionally, confocal laser scanning microscopy confirmed that GA-GO could facilitate the delivery of DOX into mitochondria of HepG2 cells since it induced the MMA of cancer cells. In addition, GA-GO@DOX caused an increase in the MMA through activation of the key cascade proteins of this pathway. This was also in vivo in HepG2-bearing BALB/c nude mice, where GA-GO@DOX exhibited an antitumor activity superior to that of free DOX.

Chopdey and coworkers investigated the drug-targeting potential of GA-conjugated polypropylene imine (PPI) dendrimers (GA-PPI) and multi-walled carbon nanotubes (GA-MWCNTs) for the liver targeting of DOX [70]. Higher release of DOX from the PPI dendrimer formulation was observed at pH 5.5 as compared to phosphate buffer (pH 7.4), which was very likely because the amine groups of PPI dendrimers undergo protonation at low pH. Additionally, drug release from MWCNTs was dependent on the pH of the environment since the drug is ionized in acidic conditions. Cytotoxicity assays on HepG2 showed, among all formulations, the minimum IC_50_ value for GA-PPI-DOX, which was found to be dragging more cells in the apoptotic phase as compared to GA-MWCNT-DOX. This effect is probably due to more conjugation of GA on the PPI periphery than on MWCNT formulations. Furthermore, the in vitro release of GA-MWCNT-DOX was slower and sustained as compared to GA-PPI-DOX.

## 8. GA-Functionalized Systems for Gene Therapy

Today, there is a great interest in next-generation therapies that use biological macromolecules, such as plasmid DNA, short interfering RNA (siRNA) or antisense nucleotides, so that new DDS techniques able to improve therapeutic efficacy by taking into account the molecular mechanisms of these new therapeutic agents are required. However, since these products are biological macromolecules, they cannot simply pass cell membranes, so it is essential to develop new technologies to protect them from nuclease degradation and allow them to be easily introduced into cells. 

CS has been shown to protect siRNA from serum degradation and deliver it to tumor cells. Zheng and coworkers [71] tested a dual receptor-targeted CS nanosystem controlled by LA and GA (GCGA). This system was loaded with siPAK1, a siRNA targeting P21-activated kinase 1 (PAK1), a downstream effector of a wide variety of mitogenic factors implicated in HCC progression and metastasis. In vitro data confirmed that GA and LA exhibited a superior targeting capacity, as demonstrated by free GA or LA competitive inhibition assay. Hep3B-xenografted BALB/c nude mice were injected with siPAK1-loaded NPs that tended to accumulate in the tumor foci rather than in normal tissues. The findings led the authors to hypothesize that GCGA-siPAK1 promotes endogenous cell apoptosis through the PAK1/MEK/ERK pathway.

Furthermore, the co-delivery of chemotherapeutic drugs and siRNA can improve antitumor efficacy compared to a single administration. Some siRNAs, such as Bcl-2 siRNA, can target the Bcl-2 gene, inhibit Bcl-2 protein synthesis, and induce apoptosis of tumor cells. CS-PEI hybrid systems have been prepared to enhance the transfection efficiency of genomic medicines. Yan and coworkers [72] tested the chemotherapeutic effects of nanomicelles based on the prodrug polymer GA-CS-PEI-HBA-DOX (DOX attached to CS-PEI through a pH-sensitive linker 4-hydrazinobenzoic (HBA), thus obtaining a bond hydrolysable at acidic pH) and Bcl-2 siRNA (GA-CS-PEI-HBA-DOX@siRNA). PEI is a polycationic vector largely employed as a gene delivery system for cancer therapy due to its cationic charge and buffering capacity, which renders it suitable to condense large negatively charged molecules, to protect DNA from degradation, and to induce endosomal escape of the gene payload. This system, GA-CS-PEI-HBA-DOX@siRNA, demonstrated superior co-delivery and anticancer targeting abilities based on the pH-responsive drug release, surface charge conversion as a function of pH values, and receptor-mediated endocytosis. In fact, positively charged NPs could be easily uptaken by cancer cells via “electrostatic attraction-mediated targeting”, since the surface of most cancer cells is maintained negative. The competitive inhibition of GA-CS-PEI-HBA-DOX@siRNA by free GA over GA receptors on HepG2 cells confirmed the active targeting properties of this DDS on hepatoma cells. In addition, the GA-CS-PEI-HBA-DOX@siRNA showed much stronger cytotoxicity to HepG2 compared to siRNA or DOX or siRNA/DOX in combination. In vivo data on HepG2-bearing BALB/c nude mice demonstrated that GA-CS-PEI-HBA-DOX@siRNA exhibited the highest therapeutic effect when compared to moderate antitumor properties of free DOX and limited inhibitory effects of siRNA. Furthermore, no effects on normal tissues were reported for the nanoformulations when compared to the serious side effects of free DOX.

The same strategy has been tested using liver-targeted NPs composed of DSPE-PEG-PEI (DPP) with GA-modified HA (GH) for the co-delivery of DOX and Bcl-2 siRNA (siRNA/DOX/GH-DPP) [73]. The half-maximal inhibitory concentrations (IC_50_ value) of siRNA/DOX/DPP and siRNA/DOX/GH-DPP NPs against HepG2 cell viability was 1.02 and 0.76 DOX µg/mL, respectively, which were lower than that of free DOX (1.86 DOX µg/mL), supporting an improved cellular uptake of DOX and siRNA via GA receptor-mediated endocytosis and the better sensitivity of HepG2 cells to DOX owing to down-regulation of Bcl-2 by RNA interference. In vivo data confirmed liver-targeting delivery and decreased uptake by normal cells of siRNA/DOX/GH-DPP in H22 tumor-bearing mice, resulting in higher anti-hepatoma efficacy than siRNA/DOX/DPP NPs and less systemic toxicity compared to free DOX.

Since HCC is associated with the activation of the PI3K/Akt/mTOR signaling pathway, facilitating the development of tumor cell proliferation, angiogenesis, metastasis, and invasion, Wang and coworkers [74] tested a nanosystem self-assembled from a PEI-GA amphiphilic copolymer as a versatile gene/drug dual delivery nanoplatform using DOX and a short hairpin RNA silencing Akt1 (shAkt1). The IC_50_ of free DOX, PEI-GA/DOX, and PEI−GA/DOX/shAkt1 NPs in HepG2 was estimated to be approximately 4.56, 2.07, and 0.99 μg/mL, respectively, confirming the higher drug delivery and the synergic activity of combination therapy. In addition, after treatment with PEI-GA/DOX/shAkt1 NPs, the reduction of Akt1 protein level in HepG2 cells induced autophagy, as an alternative pathway to cell death (type II cell death), via LC3B−II protein upregulation. Furthermore, in vivo treatment with PEI-GA/DOX/shAkt1 NPs indicated improved tumor growth inhibition in Hepa-1.6 cell grafted tumor-bearing C57BL-6J mice.

However, low-molecular-weight PEIs (LMWH, below 2000 Da) are known for their low toxicity but poor transfection. Cao and coworkers designed GA-modified LMWH PEIs to demonstrate that modification of LMWH PEIs with GA could give high transfection efficiency and allow liver targeting [75].

GO represents a potentially useful material in gene therapy due to its 2D planar structure with the high presence of surface oxygenated functional groups and due to its ability to easily cross cell membranes. To ameliorate these properties, the GO surface may be modified by positively charged cationic polymers. Polyamidoamine (PAMAM) dendrimers are biodegradable cationic and highly branched spherical polymeric macromolecules with a peptide bond backbone, and their conjugation with GO has been explored as an innovative approach for gene delivery. Liu and coworkers [76] demonstrated efficient intracellular delivery of plasmid DNA using GA-PAMAM-GO nanohybrids with good transfection efficiency in SMMC-7721 cells. More recently, GA was employed by Qu and coworkers as a liver-targeting ligand to construct GA, PEG, PAMAM dendrimer (D) and nano-graphene oxide (NGO) conjugates (GPND) for siRNA delivery targeting VEGF, a well-known pivotal regulator of tumor angiogenesis [77]. Data demonstrated efficient cell uptake of the GPND/siRNA nanocomplex and gene silencing in HepG2 cells. The mechanism involved in the effect of this system is that PAMAM, due to its proton sponge effect, can attract hydrogen ions and thus cause penetration of chloride ions and water into lysosomes, resulting in their rupture. At the same time, PAMAM dendrimers are degraded in the acidic environment, allowing effective siRNA release. Notably, in vivo studies showed an evident siRNA accumulation in liver tumor tissue by the delivery of GPND, thus leading to significant growth inhibition of tumor tissues in HepG2-bearing NUNU mice.

## 9. GA-Functionalized Biomaterials for Photodynamic Therapy

Photodynamic therapy (PDT) is a promising alternative for cancer treatment, especially due to its painless and non-invasive modalities. A photosensitizer (PS), systemically or topically administered, accumulates in the target site during a predetermined duration time (drug-to-light interval), after which the target site is irradiated by the light of appropriate wavelength and energy, producing PS photo-excitation. This excited PS has to transfer its energy to surrounding intracellular oxygen, forming cytotoxic reactive oxygen species (ROS). However, the light energy absorbed by a PS can also be released through fluorescence or heat generation. The main targets of irradiated PS-induced damage are represented by mitochondria, lysosomes, plasma membranes, nuclei, and blood vessels around cancer cells [108]. To be clinically used, PSs must be able to highly and selectively accumulate in the tumor, possess only low or minimal dark toxicity, and be characterized by high bio-stability and high bio-clearance. However, despite the great progress in PS-mediated PDT, their clinical uses are still reduced due to the poor water solubility and tissue/cell specificity of conventional PS drugs. In this regard, the development of materials that incorporate PS drugs and transfer them into target tissues/cells is required.

For example, since GA can improve the uptake of PS to cancer cells, Wang and coworkers studied the properties of the amphiphilic GA-porphyrin (TPP) conjugate self-assembled into NPs (TPP-GA NPs). TPP, one of the most widely used photosensitizers, is hydrophobic, while GA is water soluble, so the amphiphilic conjugate TPP-GA can self-assemble into NPs. In vitro experiments demonstrated that TPP-GA NPs are uptaken by endocytosis into tumoral cells, and under opportune irradiation (620 nm, 12 mW cm^−2^ for 1 h), it showed light phototoxicity in HepG2 cells when compared to cells maintained in the dark, suggesting that TPP-based nanomaterials could be applied for the PDT of cancer cells [78].

Phthalocyanine has a high absorbance at 600−700 nm, high ROS generation efficiency, and is also stable from chemical and photochemical degradation. Silicon phthalocyanine (SiPC) was linked to GA using PEG and 3-(ethoxydimethylsilyl)propylamine (APDES) [79]. GA-PEG-SiPC was internalized via GA receptor, showing significant cytotoxicity when the liver cancer HepG2 and Huh7 cells were irradiated using a 671 nm light source (50 mW cm^−2^) for 80 s. After intravenous administration in HepG2 tumor-bearing mice, GA-PEG-SiPC accompanied with PDT revealed liver cancer-targeted accumulation and anticancer properties via apoptosis and necrosis without side effects and resistance to treatment.

DDSs projected for the combinate use of chemotherapeutics and PS are an innovative strategy for cancer treatment, also because ROS produced by PS irradiation can disrupt the lysosome membrane and thus induce lysosome escape of the drugs. Then, DOX and the photosensitizer pheophorbide A (PHA) were loaded into micelles made of the poly-ε-caprolactone-cystamine-carboxymethyl CS-GA (PCL-SS-CMC-GA) polymer, where the switchable disulfide bonds were predisposed to be degraded in the high redox potential of cancer cells and trigger the release of therapeutic agents [80]. The redox-responsive release mechanism of PCL-SS-CMC-GA@DOX/PHA can allow the controlled release of drugs specifically in tumor cells. The difference can be due to the glutathione (GSH) levels, since its concentration in the extracellular matrix and body fluids of normal tissues is about 2~20 μM, but it can reach 20 mM in tumor cell endosomes with important changes in redox potential. The findings obtained in HepG2 cells showed that both the functionalization with GA and the charge conversion property of this system can promote its adsorption and uptake by tumor cells, while the cytotoxicity of the PCL-SS-CMC-GA@DOX/PHA system is dependent on laser irradiation. The biodistribution in tumor-bearing BALB/c nude mice showed that PCL-SS-CMC-GA@PHA accumulated in the tumor site when compared to GA-undecorated micelles and confirmed a stronger inhibition rate (54.1%) of the subcutaneous tumors after laser irradiation compared to the controls.

## 10. Conclusions

Chemotherapy is the main treatment for hepatic tumors, including HCC, but it is still challenging due to several problems, including its nonselective biodistribution and effects, as well as multidrug chemoresistance. Active TDDSs, based on the interaction between a ligand present on the surface of the DDS and specific receptors present on the target cells, hold enormous potential in cancer therapy for their capability to improve DS internalization and drug uptake into specific cells with higher drug bioavailability and lower systemic toxicity.

Taking this into account, our review systematically describes the more recent advances in the development of biomaterials based on GA for active drug delivery targeted to liver cancer. Besides the capability to target hepatic cells, some of these systems can offer, due to their unique chemical characteristics, several additional advantages.

First, innovative systems aimed at the simultaneous delivery of more therapeutic agents acting on different targets or mechanisms involved in cancer growth and progression have been developed. In particular, one has to remember that GA itself has well-documented anti-cancer properties. Another important aspect involves delivering nucleic acids such as siRNA/pDNA, since these therapeutic agents can enter target cells, usually via endocytosis, and their functionality strongly depends on the efficiency of endosomal escape. Particularly interesting is the development of GA-based biomaterials that incorporate PS drugs to be used in liver cancer PDT.

Special attention is focused on dual functionalization and on the design of stimuli-responsive systems. In fact, a recent trend in the surface functionalization of DDSs involves their decoration with two ligands (for example, GA together with LA), enabling them to deliver more drugs to specific cells and further reduce normal tissue toxicity. There has been increased development of GA-based systems responsive to intrinsic characteristics of tumor microenvironments, in particular the lower pH.

Thus, these results highlight the potential therapeutic efficacy of rationally designed TDDSs based on GA and lead us to hypothesize that the research about these products will continue to increase in the future. However, at this phase of research, the translation of the existing data from the laboratory to the clinical field appears to still be very problematic. A particular problem is represented by the limited scientific knowledge about the hepatic GA receptors. Although several papers have demonstrated the existence of these proteins called GA receptors on the membranes of normal and tumoral liver cells, we know very little about the physiological and biochemical aspects related to their functionality.

On the other hand, the efficacy of all the products reported in this review has been demonstrated in vitro on opportune cancer cell lines, and in a few cases, in vivo using experimental animals. Thus, the difficulty of extrapolating these experimental findings from isolated cell lines to complex biological systems and then to humans is evident, and only further studies can allow us to demonstrate their actual clinical efficacy, as well as their safety and biocompatibility.

One has also to point out that the increased complexity of methodologies employed to realize the described TDDSs can also introduce significant obstacles in reproducibility, scale-up/out, and quality control, and consequently significantly higher production costs, all aspects that make their applicability to the clinic more complex.

Finally, several obstacles have limited the clinical application of licorice-derived therapeutics. The metabolism of a large part of marketed drugs is regulated by cytochrome P450 (CYP) isoforms (CYP3A, CYP2C9, CYP2C19, CYP2D6, CYP2E1, etc.), which are most frequently involved in drug phase I biotransformation, and by uridine 5′-diphospho-glucuronosyltransferases (UGTs), which play a main role in phase II metabolism. Furthermore, the activity of the transmembrane ATP-binding cassette transporter P-glycoprotein, predominantly expressed in the intestinal tract, brain, liver, and kidney, is also crucial for drug metabolism and bioavailability. It has been reported that GA can significantly affect the activity of some metabolic enzymes, including several CYP450 isoforms and UGTs, as well as of P-gp, so it could mediate potential drug-drug interactions. In addition, a further complication due to licorice uptake seems to be pseudohyperaldosteronism, a clinical condition characterized by hypertension, hypokalemia, and suppression of plasma renin and aldosterone levels. This effect is due to its component, GA, which acts mainly through two different mechanisms: by blocking the enzyme 11-β-hydroxysteroid dehydrogenase type 2 (11-β-HSD2), which inactivates cortisol to cortisone, and by directly binding the mineralocorticoid receptor as an agonist [109].

In conclusion, active targeted biomaterials based on GA represent a great promise for the development of an innovative therapeutic strategy in the treatment of liver cancer, although there is an imperative need for further studies to demonstrate their efficacy and safety.

## Abbreviations

∆Ψm	membrane potential
11-Deo-GA	11-deoxy-glycyrrhetinic acid
3-Ace-GA	C3-acetylated glycyrrhetinic acid
3-*O*-gluc-GA	glycyrrhizic acid
5-FU	5-fluorouracil
ADE	adenine
AGP	andrographolide
aHSCs	activated HSCs
APDES	3-(ethoxydimethylsilyl)propylamine
APS	*Angelica sinensis* polysaccharide
ASGP-R	asialoglycoprotein receptor
ART	artesunate
ATP	adenosine triphosphate
Bcl-2	B-cell lymphoma 2
CA4P	combretastatin A4 phosphate
CAPS	capsaicin
CMC	carboxymethyl chitosan
CPT	camptothecin
CS	chitosan
CUR	curcumin
CYP	cytochrome P450
Cyt c	cytochrome c
DDS	drug delivery system
DEN	diethylnitrosamine
DiR	3′-tetramethylindotricarbocyanine iodide
DOX	doxorubicin
DSGPE	1,2-distearyl-sn-glycero-3-phosphoethanolamine
DSPE	distearoyl-phosphatidylethanolamine
DTX	docetaxel
EGF-R	epidermal growth factor receptor
EMA	European Medicines Agency
EPR	enhanced permeation and retention effect
ERK	extracellular signal-regulated kinase
FA-R	folate receptor
FDA	Food and Drug Administration
FITC	fluorescein isothiocyanate
Fmoc	9-fluorenylmethyloxycarbonyl
GA	glycyrrhetinic acid
Gal	galactose
Gal-β(1–3)GalNA	β-d-galactosyl-(1–3)-*N*-acetyl-d-galactosamine
GH	GA modified HA
GHH	GA-HA-Histidine
GL-R	glycyrrhizin receptor
GO	graphene oxide
GR	galactose receptor
HA	hyaluronic acid
HA-R	hyaluronan receptor
HBA	4-hydrazinobenzoic
HCC	hepatocellular carcinoma
HSCs	hepatic stellate cells
HZ	hydrazone
IP	intraperitoneal
IV	intravenous
LA	lactobionic acid
LMWH	low molecular weight
LRH-1	liver receptor homolog 1
LYVE-1	lymphatic vessel endothelial hyaluronan receptor 1
m-HSC	mouse hepatic stellate cell
MDR	multidrug resistant
MEK	mitogen-activated protein/extracellular signal-regulated kinase
MMP	matrix metalloproteinase
MOFs	metal-organic frameworks
MPT	mitochondrial permeability transition
mPEG	polyethylene glycol methyl ether
mTOR	mechanistic target of rapamycin
MR	mannose receptor
MSN	mesoporous silica nanoparticle
MWCNTs	multi-walled carbon nanotubes
NCTD	norcantharidin
NGO	nano-graphene oxide
NIR	near-infrared
NK-1R	neurokinin-1 receptor
NMOFs	nano-sized metal-organic frameworks
NP	nanoparticle
OX	oxaliplatin
PAK1	P21-activated kinase 1
PAMAM	poly(amidoamine)
PCL	poly-ε-caprolactone
PCNA	Proliferating Cell Nuclear Antigen
PDT	photodynamic therapy
PEG	polyethylene glycol
PEG2000	polyethylene glycol 2000
PEI	polyethylenimine
PHA	pheophorbide A
PHIS	poly(l-histidine)
PI3K	phosphatidyl Inositol 3-kinase
PLA	polylactic acid
PLGA	poly(d,l-lactide-co-glycolide)
PNA	peanut agglutinin
PPI	polypropylene imine
PS	photosensitizers
PTX	paclitaxel
RBCm	red blood cell membranes
ROS	reactive oxygen species
SC	subcutaneous
SH	sulfhydryl group
sHA	sulfated hyaluronic acid
shAkt1	shRNA targeting Akt1
shRNA	short/small hairpin RNA
siPAK1	siRNA-targeting P21-activated kinase 1
SiPC	silicon phthalocyanine
siRNA	short interfering RNA
SP	substance P
SS	disulfide
TDDS	targeted drug delivery system
TGF-β	transforming growth factor-β
TPP	tetraphenylporphyrin
UGTs	uridine 5’-diphospho-glucuronosyltransferases
VEGFa	vascular endothelial growth factor a
WG	wogonin
